# The Effect of Drug Heterogeneous Distributions within Core-Sheath Nanostructures on Its Sustained Release Profiles

**DOI:** 10.3390/biom11091330

**Published:** 2021-09-09

**Authors:** Haixia Xu, Xizi Xu, Siyu Li, Wen-Liang Song, Deng-Guang Yu, S. W. Annie Bligh

**Affiliations:** 1 School of Materials Science and Engineering, University of Shanghai for Science and Technology, Shanghai 200093, China; 193742716@st.usst.edu.cn (H.X.); 192432592@st.usst.edu.cn (X.X.); 1826410102@st.usst.edu.cn (S.L.); wenliang@usst.edu.cn (W.-L.S.); 2 Shanghai Engineering Technology Research Center for High-Performance Medical Device Materials, Shanghai 200093, China; 3 School of Health Sciences, Caritas Institute of Higher Education, Hong Kong 999077, China

**Keywords:** sustained release, water-soluble drug, core–sheath structures, triaxial electrospinning, cellulose acetate, metformin hydrochloride

## Abstract

The sustained release of a water-soluble drug is always a key and important issue in pharmaceutics. In this study, using cellulose acetate (CA) as a biomacromolecular matrix, core-sheath nanofibers were developed for providing a sustained release of a model drug—metformin hydrochloride (MET). The core–sheath nanofibers were fabricated using modified tri-axial electrospinning, in which a detachable homemade spinneret was explored. A process—nanostructure–performance relationship was demonstrated through a series of characterizations. The prepared nanofibers F2 could release 95% of the loaded MET through a time period of 23.4 h and had no initial burst effect. The successful sustained release performances of MET can be attributed to the following factors: (1) the reasonable application of insoluble CA as the filament-forming carrier, which determined that the drug was released through a diffusion manner; (2) the core–sheath nanostructure provided the possibility of both encapsulating the drug completely and realizing the heterogeneous distributions of MET in the nanofibers with a higher drug load core than the sheath; (3) the thickness of the sheath sections were able to be exploited for further manipulating a better drug extended release performance. The mechanisms for manipulating the drug sustained release behaviors are proposed. The present proof-of-concept protocols can pave a new way to develop many novel biomolecule-based nanostructures for extending the release of water-soluble drugs.

## 1. Introduction

Sustained release, one of the most popular and common release profiles, refers to the slow release of the loaded drugs from its dosage forms in the specified release medium after oral administration [1,2,3,4,5]. Compared with the fast release dosage forms, the administration frequency can be reduced, and, thus, the sustained release profile can significantly increase the patient’s compliance [6,7,8,9]. Meanwhile, the sustained release of the drug can also effectively keep the drug blood concentration from being a toxic value, and correspondingly reduce the possible side effects of the drug [10,11,12,13].

During the past several decades, various raw materials and techniques have been expanded into the field of drug delivery, an interdisciplinary application field [14,15,16,17]. Among all sorts of raw materials (such as inorganic nanoparticles, carbon nano tubes, graphene, and organic molecules) [18,19,20,21], biomacromolecules, which often have a natural source, have received increased attention due to their fine biocompatibility, low toxicity, abundance in nature, and, often, their ease of processing [22,23,24,25,26]. An excellent example is cellulose, the most applied polymer in the modern industry [27,28,29]. Cellulose acetate (CA), first prepared in 1865, is the acetate ester of cellulose. CA is a chemically modified polymer compound obtained by the esterification of hydroxyl groups in cellulose molecules with acetic acid [30,31,32]. In traditional pharmaceutics, CA is frequently utilized for preparing enteric coating, acetate fiber filter membrane, and a wide variety of drug sustained release dosage forms, such as tablets, foam, capsules, pellets, and also medicated implants [33,34].

In this nano era, more and more nanotechnologies are being introduced into pharmaceutics as new nanopharmaceutical methods [35,36,37,38,39,40]. All nano methods that are explored to prepare nanoparticles, nanofibers, and nanosheets can find their potential applications in creating new sorts of nano drug delivery systems [41,42,43]. Electrospinning, as a brother method of electrospray, is just an example that shows this trend [44,45,46,47]. Electrospinning was revived around 1995, and, in 2002, the first drug delivery investigation was reported, which simply concerned the drug sustained release from electrospun nanofibers [48,49]. For improving the drug sustained release profile, the after-treatment of cross-linking was exploited on those electrospun nanofibers. With the progress made of electrospinning itself, more and more strategies have been developed for furnishing all types of drug controlled release profiles, including pulsatile, biphasic, targeted, and drug sustained release [50,51,52,53,54].

The sustained release of drugs from their electrospun nanofibers has developed into a strong branch today. Different types of electrospinning methods, new kinds of filament-forming polymeric matrices, and composites and hybrids comprising both polymers and inorganic nanoparticles are successively reported [55,56]. In literature, most of them are centered around the poorly water-soluble drugs [57,58,59]. In contrast, the electrospun nanofibers and other kinds of products that also provide a sustained release profile of water-soluble drugs are very limited [60,61,62], which is also a key and difficult issue in the pharmaceutical field [63,64].

MET has the effect of lowering the blood pressure, blood fat, and weight control. It is the first choice for type II-diabetes with obesity and insulin resistance. It has a synergistic effect with insulin or sulfonylureas oral hypoglycemic agents, and can be used for the treatment of patients with insulin resistance, and can also be used for the treatment of polycystic ovary syndrome and non-alcoholic fatty liver disease [65,66]. It is well known that the sustained release of MET is highly desired by the patients, and some commercial products can be found in markets [67,68,69,70,71,72].

Based on the above-mentioned background, in the present work, a new kind of core–sheath CA nanofiber is fabricated for providing a sustained release of metformin hydrochloride (MET), a popular water-soluble drug model [63,64]. Modified tri-axial electrospinning was conducted for producing the core–sheath nanofibers, in which an additional outer solvent was exploited for a smooth and robust preparation [73,74].

## 2. Experimental

### 2.1. Materials

Both MET and CA (*M*_W_ = 100,000, with an acetylation degree of 39.8%) were obtained from Sigma-Aldrich Co., Shanghai, China. The organic solvents, including acetone, *N*,*N*-dimethyl acetamide (DMAc), and ethanol, were purchased from the Shanghai 1st Shi-Ji Factory. Color markers for optimizing the working processes, i.e., basic fuchsin and methylene blue, were bought from Sinopharm Chem. Co., Shanghai, China. Water was doubly distilled. All chemicals were of analytical grade.

### 2.2. Electrospinning

Three spinning solutions were prepared for implementing the tri-axial electrospinning. A mixture comprising acetone, DMAc, and ethanol in a volume ratio of 4:1:1 was exploited for preparing all three solutions. A ratio of 3% and 8% (*w*/*v*) MET was dissolved into the solvent mixture for preparing the middle and inner fluids, respectively. The outer fluid was the pure solvent mixture. The distance between the collector and the tip of spinneret was 20 cm during all the preparations. Two kinds of core–sheath fibers (denoted as F1 and F2) were prepared with various middle-core fluid flow rates. Meanwhile, two homogeneous nanofibers (denoted as F3 and F4) were prepared from the modified coaxial processes. The key experimental parameters are included in Table 1.

The homemade tri-axial spinning system consisted of a detachable three-layer concentric spinneret, a ZGF 60 kV/2 mA high-power supply (Wuhan Hua-Tian Corp., Wuhan, China), a fiber collector, and three fluid drivers (KDS100, Cole-Parmer, Vernon Hills, IL, USA). The environmental temperature was 21 ± 4 °C, and the humidity was 47 ± 5%. All of the prepared nanofibers were stored in a vacuum drying oven to reach a constant weight before characterizations.

### 2.3. Characterization

#### 2.3.1. Morphology and Structure

A field emission scanning microscope (SEM, Hitachi, Tokyo, Japan) was utilized to assess the nanofibers’ morphology. The fiber samples were gold sputter-coated for 1 min under nitrogen atmosphere. The nanofibers’ structures were evaluated through a transmission microscope (TEM, JEM2100F, JEOL, Tokyo, Japan).

#### 2.3.2. Physical State and Compatibility

An X-ray diffractometer (XRD, Bruker-AXS with Cu Kɑ radiation, Karlsruhu, Germany) was utilized to measure the physical state of samples from 5° to 60° of 2θ under a voltage and current of 40 kV and 30 mA, respectively. The scan speed was 0.02 degree/s. A Spectrum 100 spectrometer (PerkinElmer, Billerica, MA, USA) was exploited to conduct the Fourier-transform infrared (FTIR) spectroscopy from 500 cm^−1^ to 4000 cm^−1^, with a resolution of 1 cm^−1^ to disclose the compatibility of components within the nano products.

#### 2.3.3. Drug Encapsulation Efficiency and In Vitro Dissolution Tests

A standard equation for measuring MET concentration in the solution was built using several solutions with predetermined concentrations from 1.0 to 20.0 μg/mL, which was *A* = 0.07589*C* + 0.02491 (R = 0.9998) under the maximum absorbance at λ_max_ = 234 nm, where *A* and *C* (μg/mL) represent absorbance and drug concentration, respectively. A UV-2102PC UV-vis spectrophotometer (Unico Instrument Co., Shanghai, China) was exploited.

For determining the drug encapsulation efficiency (*EE*, %), 50.0 mg medicated nanofibers were dissolved into 10.0 mL DMAc to free all the loaded MET. Then, 1.0 mL of the solution was dripped into 500 mL phosphate buffer saline (PBS, pH7.0, 0.1M). After scanning, the content of MET (*C_M_*) can be calculated from the standard equation. Meanwhile, the theoretical value of MET (*C_T_*) in the nanofibers can be calculated from the preparations of spinning solutions. Thus, EE of the nanofibers can be determined as follows:

(1) $$EE(\%)=\frac{C_{M}}{C_{T}}\times 100\%$$

In vitro drug release studies were performed using the basket method in the Chinese Pharmacopoeia (2020 Ed.). Nanofibers were placed into the baskets, which were then introduced in 600 mL of PBS (pH 7.0, 0.1M) contained in 900 mL vessels of the RCZ-8A dissolution apparatus (Tianjin University Radio Factory, Tianjin, China). The drug release studies were performed at 37 ± 0.5 °C and at a rotation rate of 50 rpm. At preset time intervals, an aliquot of 5 mL of the dissolution media was withdrawn and 5.0 mL fresh PBS was added to the vessels to keep a constant total volume of dissolution medium. The absorbance at λ_max_ = 234 nm of the withdrawn aliquot (or after a suitable dilution) was measured, and the concentration can be calculated through the standard equation. Kinetics of the drug release are disclosed using the zero-order and Peppas models.

## 3. Results and Discussion

### 3.1. Electrospinning

**Figure 1:**
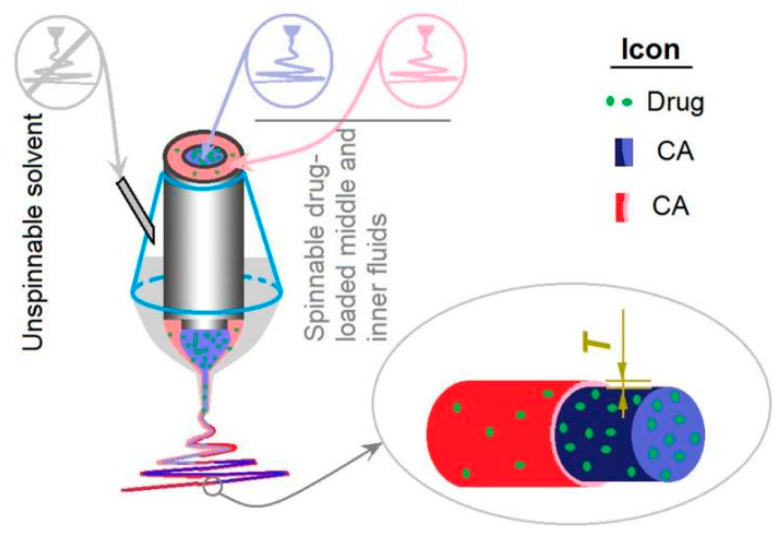
A schematic of the modified tri-axial electrospinning, in which unspinnable solvent is explored as an outer fluid for the smooth preparation of core–sheath nanofibers. *T* means thickness of the sheath section.

Exhibited in Figure 1 is a schematic about the modified tri-axial electrospinning, which is an updated process of the modified coaxial electrospinning. It is characterized by the unspinnable outer fluid, which comprises a pure organic solvent mixture. The outer solvent not only modifies the whole preparation process by changing the polymer fluid–atmosphere interfaces to solvent–atmosphere interfaces, but also improves the stability and robustness of the working processes for creating high-quality nanofibers.

In this study, both the middle and the inner fluids have fine electrospinnability, whereas the outer solvent is unspinnable. Meanwhile, the core and middle fluids are highly compatible due to the difference between them only being their drug concentration. Their compatibility results from the small interfacial tensions between the spinning solutions at different layers and the impossibility of coagulations of solutes at the fluid–fluid boundaries. Thus, it is very convenient to adjust the middle-to-core fluid flow rate ratios, which can be exploited for manipulating the thickness of the sheath layer, i.e., the *T* value in Figure 1.

**Figure 2:**
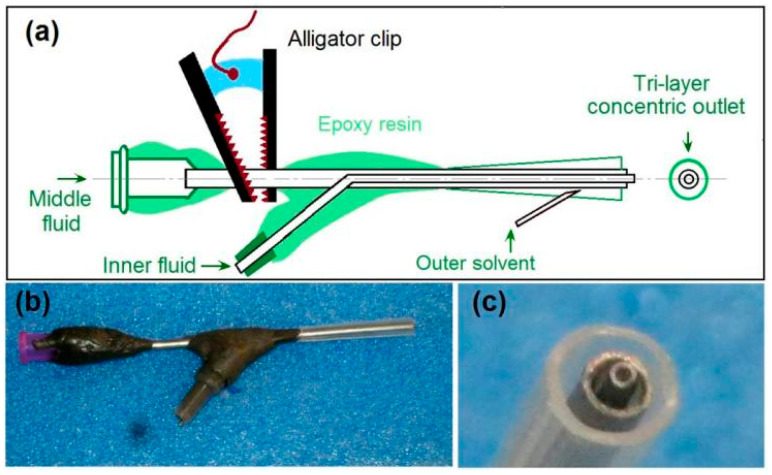
The homemade spinneret for implementing the modified tri-axial electrospinning: (**a**) the details about the inner structures of spinneret and its components; (**b**) a digital photo of the whole spinneret; (**c**) a digital photo of the tri-layer concentric outlet for leading the outer–middle–inner spinning solutions.

It is common sense that an electrospinning system often has four components (i.e., fluid driver, power supply, collector, and spinneret), and that the most important part is the spinneret. A good spinneret not only guides the different kinds of spinning solutions to the electric fields for forming the designed nanofibers, but should also be convenient for setting up the whole electrospinning system and be able to save energy [45]. In this study, a homemade spinneret is shown in Figure 2, in which a tri-layer concentric outlet for organizing the outer–middle–inner fluids is diagrammed in Figure 2a. The middle fluid inlet has a standard groove for direct connection with the syringe holding the working fluid, which can be fixed on a syringe pump and, thus, the whole spinneret can be fixed. The inner fluid and outer solvent can be led to the spinneret through silicon tubes. All of the connecting places are sealed using epoxy resin. Only a small section of the metal capillary guiding the middle fluid is left outward for the alligator clip. This special arrangement is useful for keeping the electrostatic energy from escaping to the environment and, thus, for saving energy. This point is very important for cutting the cost of the nanofibers when the production is on an industrial scale.

Figure 2b shows a digital photo of the spinneret, which has a weight of 48.2 g and, thus, is convenient for installing the electrospinning system and for prevent the falling of the spinneret from the syringe. Figure 2c is a digital photo of the three-layer outlet for leading the outer–middle–inner three spinning solutions in a concentric manner. The inner metal capillary is slightly projected out of the middle metal capillary, and, in turn, the middle metal capillary is slightly projected out of the outer polypropylene (PP) horn tube. The small end of the PP horn tube can directly be fixed to the tube on the middle metal capillary, and the large end is utilized to form the designed tri-layer concentric outlet.

**Figure 3:**
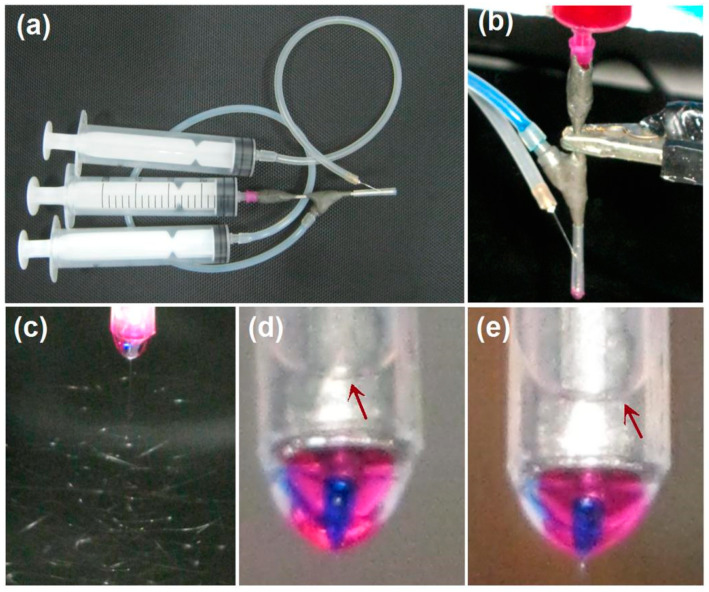
The implementations of the modified 3-axial electrospinning: (**a**) a digital photo of the linkages of three syringes with the three inlets of spinneret; (**b**) a digital photo exhibiting the pumping of three spinning solutions and the connection of a clip for transferring the electric energy from the power supply to the the spinning solutions; (**c**) a digital image showing the whipping and binding processes for preparing nanofibers F1; (**d**,**e**) the tri-layer complicated Taylor cones for preparing nanofibers F1 and F2, respectively. The red arrows indicate the sheath liquid arches.

Some important details for implementing the modified tri-axial electrospinning are concluded in Figure 3. Figure 3a shows a digital photo of the connections of three syringes with the three inlets of the spinneret. For the outer solvent mixture, a metal needle is required to penetrate the PP tube for transferring it to the outlet. Figure 3b is a photo of the working process, which was conducted for optimizing the working conditions. The electrostatic energy was converted to the spinning solutions through the alligator clip, which clamped on the only exposed metal section of spinneret.

Under the selected experimental parameters, an ongoing electrospinning process for preparing nanofibers F1 is exhibited in Figure 3c. Obviously, the three successive steps can be recognized, i.e., the compound Taylor cone (also shown in a magnified manner in Figure 3d), the straight jet, and, later, the bending and whipping instable region. As for the preparation of nanofibers F2, the digital photo of the tri-layer compound Taylor cone is exhibited in Figure 3e. It is obvious in both Figure 3d,e that the outer fluids of the solvent mixture are transparent and form an arch liquid level in the PP tube (as indicated by the red arrows). Meanwhile, the inner blue spinnable fluids (marked by methylene blue) were always surrounded by the middle red fluids (marked by the basic fuchsin). Occasionally, the core blue marker was observed escaping to the sheath fluids. However, the whole working process was stable and robust when the middle and inner fluids’ flow rates were adjusted suitably, as indicated by the Taylor cones of nanofibers F1 and F2.

### 3.2. Nanofibers’ Morphologies and Structural Characteristics

**Figure 4:**
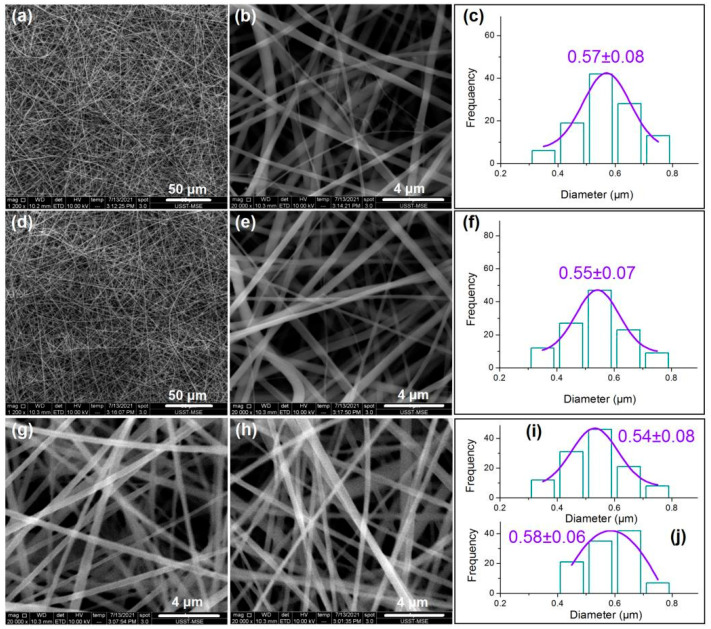
The SEM images of the four sorts of nanofibers: (**a**,**b**) nanofibers F1 under different magnifications; (**c**) diameter distribution of nanofibers F1; (**d**,**e**) nanofibers F2 under different magnifications; (**f**) diameter distribution of nanofibers F2; (**g**) nanofibers F3; (**h**) nanofibers F4; (**i**,**j**) diameter distributions of nanofibers F3 and F4, respectively.

The SEM observations of the four types of fibers and their diameters’ distributions are concluded in Figure 4. From Figure 4a to Figure 4b,c, it is clear that nanofibers F1 are linear without any spindles-on-a-line or beads-on-a-line phenomena. These nanofibers have an average diameter of 570 ± 80 nm. Similarly, nanofibers F2 have a linear morphology with a size distribution of 550 ± 70 nm, as indicated from Figure 4d to Figure 4e,f. These core–sheath nanofibers are heterogeneous materials due to their sheath and core sections having different compositions, although their components are the same in CA and MET.

For comparison, nanofibers F3 and F4 were prepared using modified coaxial electrospinning. Nanofibers F3 were produced by stopping the supply of the inner fluid while the flow rate of the middle fluid was elevated to 2.0 mL/h. Similarly, nanofibers F4 were produced by switching off the middle fluid while the flow rate of the inner fluid was increased to 2.0 mL/h. Shown in Figure 4g,i for nanofibers F3, and Figure 4h,j for nanofibers F4, these nanofibers are similarly linear in morphology without discerned spindles or beads, and they have similar diameters and distributions: 540 ± 80 nm and 580 ± 60 nm for nanofibers F3 and F4, respectively. These nanofibers have both the same components and the same compositions, and, thus, they are homogeneous nanomaterials.

**Figure 5:**
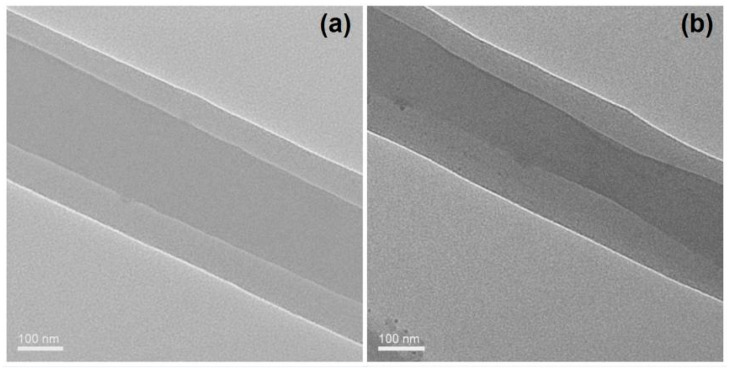
TEM images of the nanofibers: (**a**) nanofibers F1; (**b**) nanofibers F2.

The TEM images of the prepared nanofibers F1 and F2 are included in Figure 5a,b, respectively. It is obvious that both of them have the core–sheath nanostructures. The core sections having larger gray levels should be attributed to two reasons. One is the bigger thickness in the core section than the sheath section. The other, and main, reason, is that the core section had a larger drug loading. The increased drug loading can ensure more drug molecules filled in the voids among the physical entanglements of the polymeric chains. Thus, it can be deduced that the core sections have a larger density than the sheath section. Just as anticipated, nanofibers F2 have a larger sheath thickness than nanofibers F1 due to their bigger fluid flow rate ratio of 1.2/0.8, compared to 0.8/1.2. The estimated values of the sheath thickness for fibers F1 and F2 are 90 nm and 130 nm, respectively.

Based on the measured sizes of the nanofibers’ and their cores’ diameters (*r_f_* and *r_c_*, respectively), and also the experimental conditions (including the fluid rates of core *Q*_c_ and sheath *Q*_c_, respectively; drug concentration in core and sheath fluids *C_MET-c_ and C_MET-s_*; respectively; and CA concentration *C_CA_*), the densities of whole nanofibers (*ρ_f_,* the subscript “*f*” indicates parameters for nanofibers), core sections (*ρ_c_*), and sheath sections (*ρ_s_*) can be calculated according to Equations (2)–(4), respectively:

*ρ_f_ = m/V_f_ = m/LS = Q(C_MET_ + C_CA_)/LS = Q(C_MET_ + C_CA_)/L(r_f_*^2^*π)* (2)

For the core sections of the core–sheath nanofibers*:*

*ρ_c_ = m/V = m/LS = Q_c_(C_MET-c_ + C_CA_)/LS_c_ = Q_c_(C_MET-c_ + C_CA_)/L(r_c_*^2^*π)* (3)

For the sheath sections of the core–sheath nanofibers*:*

*ρ_s_ = m/V = m/LS = Q_s_(C_MET-s_ + C_CA_)/LS_s_ = Q_s_(C_MET-s_ + C_CA_)/(r_f_*^2^*π − r_c_*^2^*π)* (4)

where *V*, *L*, and *S* represent volume, length, and surface area of the cross-section of nanofibers, respectively. Thus, the relative densities (*ρ_r_*) of the core-to-sheath sections of nanofibers F1 and F2 can be estimated according to Equation (5) as follows:

*ρ_r_ = ρ_c/_ρ_s_ = [Q_c_(C_MET-c_ + C_CA_)/L(r _c_*^2^*π)]/[Q_s_(C_MET-s_ + C_CA_)/(r_f_*^2^*π − r _c_*^2^*π)]* *= [Q_c_(C_MET-c_ + C_CA_)((r_f_*^2^*-r _c_*^2^*)]/[Q_s_(C_MET-s_ + C_CA_)r _c_*^2^*]* (5)

Then, the relative density for nanofibers F1 (*ρ_r1_*) is

*ρ_r_*_1_ = [1.2(8% + 13%)(0.285^2^ − 0.195^2^)]/[0.8(3% + 13%)0.195^2^] = 2.2367

and the relative density for nanofibers F2 (*ρ_r2_*) is

*ρ_r_*_1_ = [0.8(8% + 13%)(0.275^2^ − 0.145^2^)]/[1.2(3% + 13%)0.145^2^] = 2.2723

Based on the similar values of *ρ_r_*_1_ and *ρ_r_*_2_ from different preparation processes, it can be concluded that: (1) the more the drug is loaded, the greater the density of the drug–polymer composites; (2) the changes in fluid flow rates have little influence on the the density of the drug–polymer composites; (3) the spinning solutions’ components and compositions are the key elements in determining the nanofibers’ densities, rather than the experimental conditions.

### 3.3. Compatibility between the Drug and Carrier

**Figure 6:**
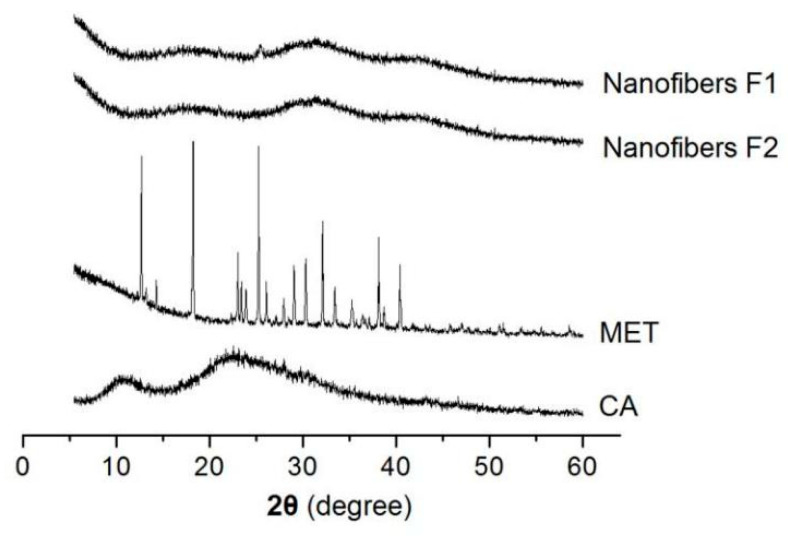
XRD patterns of CA, MET, and their core–sheath nanofibers F1 and F2.

In the medicated nanomaterials, the drug’s physical state and its compatibility with the polymeric carrier are very important for functional applications. XRD patterns are often measured to detect the physical state of the components. In this study, the XRD patterns of CA, MET, and their core–sheath nanofibers F1 and F2 are presented in Figure 6. As a crystalline material, the raw MET powders have many sharp peaks in their XRD patterns. On the contrary, raw CA powders show no peaks, except two halos, suggesting an amorphous polymer. In the XRD patterns of nanofibers F1 and F2, almost all of the sharp peaks of the raw MET disappear, giving a hint that the MET loaded in the nanofibers have lost their original crystalline state and have formed an amorphous drug–polymer nanocomposite.

**Figure 7:**
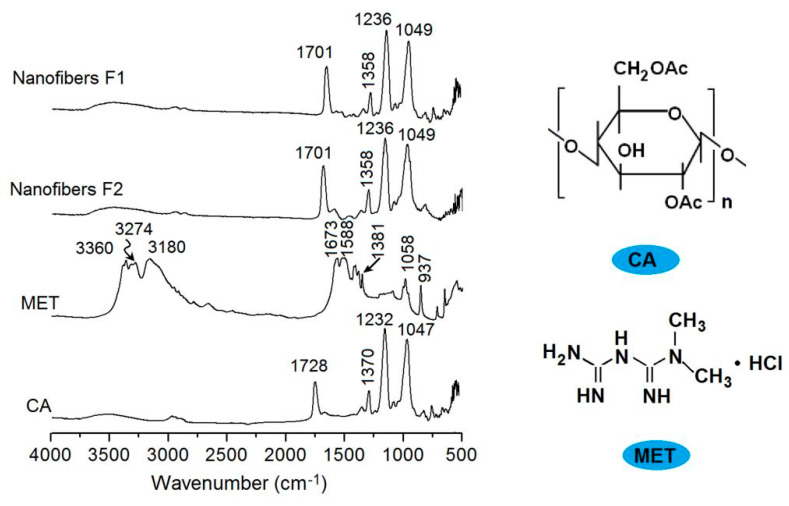
FTIR spectra of CA, MET, and their core–sheath nanofibers F1 and F2, and the molecular formula of CA and MET.

The ATR-FTIR spectra of MET, CA, and their core–sheath nanofibers are shown in Figure 7, in which the molecular formula of CA and FA are also given. Raw CA powders have characteristic peaks at 1728, 1230, 1232, and 1047 cm^−1^. Raw MET powders have characteristic peaks at 3360, 3274, 1673, 1588, 1381, and 1058 cm^−1^. However, in the core–sheath nanofibers F1 and F2, almost all of the MET peaks decreased to the point of almost disappearing. These nanofibers mainly exhibit the peaks of the polymer matrix, with some peaks slightly red shifted to a low wavenumber. These phenomena suggest that the secondary interactions happened between the CA and MET molecules. For example, the CA molecule has many —C=O groups in the —OAc groups as proton acceptors, whereas the MET molecules have —NH and —NH_2_ to act as proton donors. Thus, hydrogen bonding can be easily formed between them, and determine that CA and MET are highly compatible, and that the core–sheath nanofibers F1 and F2 are amorphous composites.

### 3.4. Drug Encapsulation Rate and Sustained Release Profiles

The measured MET loading in the nanofibers F1 and F2 were 31.1 ± 3.1% and 28.3 ± 2.9%, respectively (*n* = 3). The theoretical loading of nanofibers F1 and F2 are 31.6% and 27.8%, respectively (Table 1). Thus, the drug EEs of nanofibers F1 and F2 are 98.41% and 101.8%, respectively, suggesting almost no drug loss during the modified tri-axial electrospinning processes. Electrospinning, regardless of the working fluid numbers and regardless of spinnable fluids or unspinnable solvents, is mainly a physical drying process due to the strong interaction between the spinning solutions with the high electrostatic energy. Due to the fact that the drying processes are extremely fast (often several decades of milliseconds), the loaded drug molecules in the spinning solutions have little opportunity to escape from the jetting fluids. Their final destiny is inevitably the solidified nanofibers after electrospinning.

Apparently, this is an advantage of electrospinning for creating medicated nanomaterials because many other nano pharmaceutical methods have a low drug EE that is even smaller than 50%.

**Figure 8:**
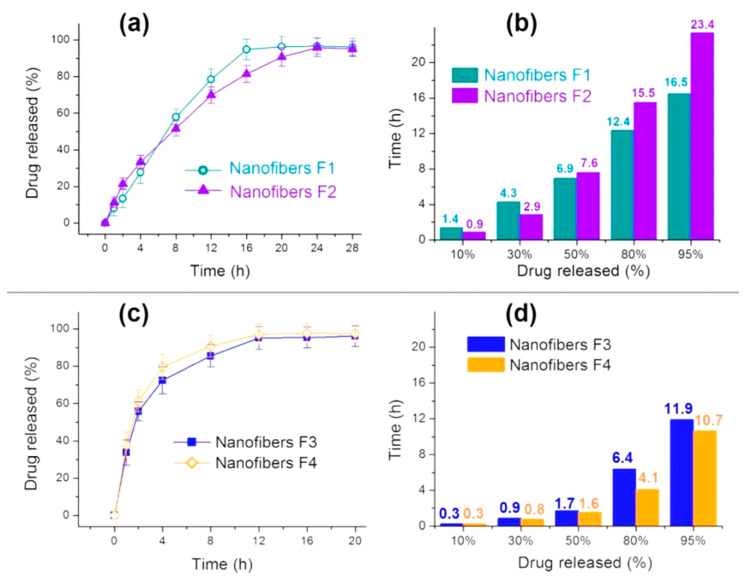
In vitro drug dissolution results: (**a**) drug release profiles of fibers F1 and F2 according to time; (**b**) the time needed for releasing a certain content of MET from fibers F1 and F2; (**c**) drug release profiles of fibers F3 and F4 according to time; (**d**) the time needed for releasing a certain content of MET from fibers F3 and F4.

The in vitro drug release profiles of nanofibers F1 and F2 are shown in Figure 8a (drug release percentage vs. dissolution time) and Figure 8b (the needed time vs. a certain percentage of the loaded drug). Both of them show an almost zero-order drug release profile, as indicated by the regressed equation in Table 2, which should be attributed to the core–sheath nanostructure and the heterogeneous drug distributions in the sheath and core sections. These drug sustained release advantages are clearer when compared with the homogeneous nanofibers F3 and F4 in Figure 8c (drug release percentage vs. dissolution time) and Figure 8d (the needed time vs. a certain percentage of the loaded drug), which are far from the zero-order kinetics (Table 2).

The most ideal sustained release profile is the zero-order release or linear release, which means that both the initial burst effect and the late leveling-off phenomena disappear. Compared with homogeneous nanofibers F3 and F4, prepared from the middle and inner spinning solutions individually, the core–sheath heterogeneous nanofibers F1 and F2 have almost no initial burst release and very weak tailing-off effects. A comparison between nanofibers F1 and F2 suggests that the nanofibers F2 with a thicker sheath of 130 nm are able to provide a greater sustained release effect than the nanofibers F1 with a sheath thickness of 90 nm. This can be judged from Figure 8b: the nanofibers F2 can provide 23.4 h for the sustained release of 95% of the loaded MET, which is longer than the 16.5 h for nanofibers F1. It is worth noting that the drug loading amount can also exert some influences on the drug release behaviors. As suggested from Figure 8d, nanofibers F4 with a relatively higher drug loading of 38.1% had a shorter sustained release time period of 10.7 h than nanofibers F3, with a drug loading of 18.8%, which could provide a sustained release time period of 11.9 h.

To further investigate the influences of core–sheath structures on the drug molecule release behaviors, the controlled release mechanisms are discussed. The well-known Peppas equation [75] *Q* = *kt^n^* is frequently utilized to tell the truth about drug molecules free from their carriers in the dissolution media. In the equation, *Q* and *t* denote the accumulative drug release amount (%) and release time, respectively, *k* is a constant, and *n*, as the release exponent, can be exploited to indicate the drug release mechanism.

**Figure 9:**
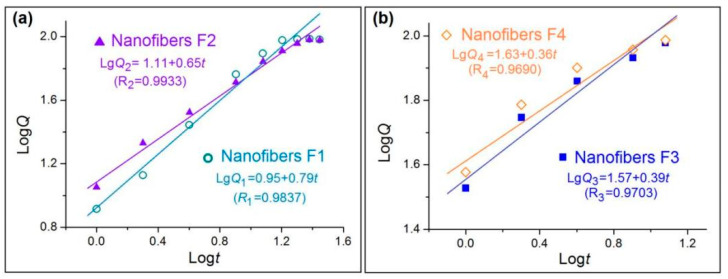
Drug release mechanisms from nanofibers F1 and F2 (**a**) and F3 and F4 (**b**).

After treatments of the in vitro dissolution test data of the four nanofibers, the regressed Peppas equations for nanofibers F1 to F4 are Lg*Q*_1_ = 0.95 + 0.79*t* (*R*_1_ = 0.9837), Lg*Q*_2_ = 1.11 + 0.65*t* (*R*_2_ = 0.9933), Lg*Q*_3_ = 1.57 + 0.39*t* (*R*_1_ = 0.9703), and Lg*Q*_4_ = 1.63 + 0.36*t* (*R*_1_ = 0.9690), respectively (Figure 9). For the homogeneous nanofibers F3 and F4 in Figure 9b, an *n* value of 0.39 and 0.36 indicate that the drug released from the nanofibers are typically controlled by the Fickian release mechanism [75], i.e., the drug molecules were released through a gradual diffusion manner. These cases have been broadly reported in the literature. However, an *n* value of 0.79 and 0.65 for core–sheath heterogeneous nanofibers F1 and F2 (Figure 9a) suggests that the drug release from them is controlled by a combination of erosion and diffusion mechanism. Due to the fact that CA is an insoluble polymer, it is impossible for the drug to be released by the erosion of the drug carrier. The reason for this contradiction should be that the Peppas equation is only suitable for disclosing the drug release mechanism from homogeneous matrices, but is inappropriate for heterogeneous products with complicated structural characteristics. The drug release from the core–sheath nanofibers F1 and F2 should be a combination of two separate diffusion stages.

### 3.5. Combined Strategy for Providing Sustained Release of Water-Soluble Drug

In traditional pharmaceutics, the sustained release for water-soluble drugs frequently relies on hydrophobic matrices, such as insoluble polymers, lipids, and even inorganic materials [76,77]. CA, as a well-known insoluble pharmaceutical excipient, has been broadly reported to furnish drug sustained release profiles in a wide variety of formats. However, the insoluble property of CA and also other cellulose derivatives is the main contributing factor for achieving the sustained release effects [78,79].

**Figure 10:**
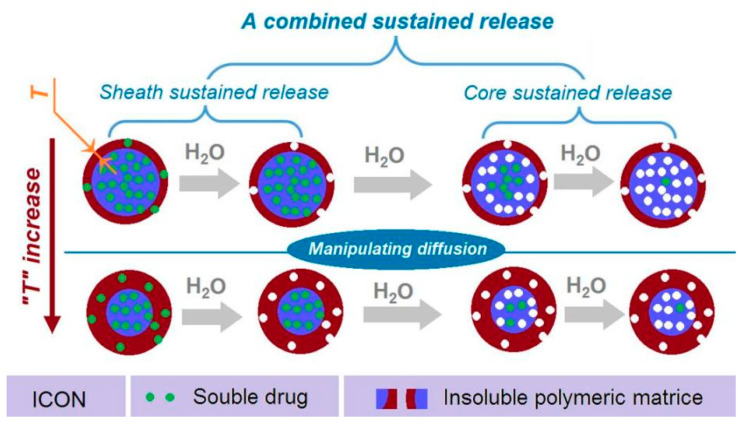
A diagram showing the combined strategy, which is aimed for providing a better sustained release profile of water-soluble drug.

In the present study, a new strategy is demonstrated for further manipulating drug sustained release behaviors. Shown in Figure 10 is a diagram of the new strategy. This strategy has made three elements act synergistically. Firstly, the reasonable selection of insoluble CA as the filament-forming matrix, which has a fine compatibility with the soluble drug MET to form a fourth generation solid dispersion, and determines that the drug is released through a diffusion mechanism. Secondly, the core–sheath nanostructure provides the possibility of both encapsulating the drugs and realizing that the heterogeneous distributions of MET in the nanofibers with a high drug-loaded core is surrounded by a lower drug-loaded sheath. By these arrangements, the typical abnormal initial burst effects can be completely eliminated. Thirdly, the thicknesses of the sheath sections (“*T*” in Figure 10) can be exploited for further manipulating a greater drug extended release performance, such as prolonging the drug sustained release time period while decreasing the tailing-off phenomena.

Oral administration is the most favorable route for the patients [80]. Electrospinning is rapidly moving to the creation of nanofibers on a large scale [81]. Based on these two aspects, the medicated nanofibers developed in the present study can be further converted to oral dosage forms for potential commercial applications, such as tablets and capsules [82,83].

## 4. Conclusions

A sustained release sheath and a sustained release core can be combined in the core–sheath nanofibers for synergistic actions in order to provide better drug sustained release profiles for the water-soluble drug MET. A modified tri-axial electrospinning process was developed for the smooth and robust preparation of the core–sheath nanofibers, in which both the core and sheath comprise CA and MET, but the core sections had a higher drug concentration than the sheath sections. SEM results demonstrated that all of the nanofibers had a fine linear morphology due to the electrospinnability of CA solutions. TEM results showed nanofibers F1 and F2 had the core–sheath nanostructures, owing to a higher density of core parts. XRD and FTIR tests suggested that CA and MET were compatible and MET presented in the CA matrices in an amorphous state. All the nanofibers had an EE of around 100%, suggesting no drug loss during the preparation processes. In vitro dissolution tests verified that the matrix CA, the core–sheath structures with different drug concentrations, and also the engineered sheath thicknesses were able to work together in a clearly combined mechanism to ensure a fine sustained release file from nanofibers F2, which had an initial release of 11.3 ± 2.9% in the first hour, released 95% of the loaded MET in 23.4 h, and had a very small late leveling-off release. The present proof-of-concept protocols can pave a new way to develop many novel biomolecule-based nanostructures for extending the release of water-soluble drug molecules.

## Figures and Tables

**Table 1:** Process parameters and theoretical drug loading of MET in the nanofibers.

No.	Spinning	Applied Voltage (kV)	Pumping Rate (mL/h)			Topography and Structure	Drug Loading ^c^ (wt%)
			Outer	Middle ^a^	Core ^b^		
F1	Triaxial	15	0.5	0.8	1.2	Linear/Heterogeneous	31.6%
F2	Triaxial	15	0.5	1.2	0.8	Linear/Heterogeneous	27.8%
F3	Coaxial	15	0.5	2.0	--	Linear/Homogeneous	18.8%
F4	Coaxial	15	0.5	--	2.0	Linear/Homogeneous	38.1%

^a^ A ratio of 3% (*w*/*v*) MET and 13% (*w*/*v*) CA were dissolved into the solvent mixture for preparing the middle fluid. ^b^ A ratio of 8% (*w*/*v*) MET and 13% (*w*/*v*) CA were dissolved into the solvent mixture for preparing the inner fluid. ^c^ Theoretical drug loading of MET calculated according to the experiemntal conditions.

**Table 2:** Kinetics of the drug release regressed according to the zero-order and Peppas models.

No.	Zero-Order		Peppas	
	Equation	*R*	Equation	*R*
F1	*Q*_1_ = 6.07 + 5.21*t*	0.9782	Lg*Q*_1_ = 0.95 + 0.79*t*	0.9837
F2	*Q*_2_ = 12.39 + 3.96*t*	0.9737	Lg*Q*_2_ = 1.11 + 0.65*t*	0.9933
F3	*Q*_3_ = 32.07 + 4.96*t*	0.8486	Lg*Q*_3_ = 1.57 + 0.39*t*	0.9703
F4	*Q*_4_ = 36.23 + 4.89*t*	0.8164	Lg*Q*_4_ = 1.63 + 0.36*t*	0.9690

## Data Availability

The data supporting the findings of this manuscript are available from the corresponding authors upon reasonable request.
